# Parcellation of the Healthy Neonatal Brain into 107 Regions Using Atlas Propagation through Intermediate Time Points in Childhood

**DOI:** 10.3389/fnins.2016.00220

**Published:** 2016-05-19

**Authors:** Manuel Blesa, Ahmed Serag, Alastair G. Wilkinson, Devasuda Anblagan, Emma J. Telford, Rozalia Pataky, Sarah A. Sparrow, Gillian Macnaught, Scott I. Semple, Mark E. Bastin, James P. Boardman

**Affiliations:** ^1^MRC Centre for Reproductive Health, University of EdinburghEdinburgh, UK; ^2^Department of Radiology, Royal Hospital for Sick ChildrenEdinburgh, UK; ^3^Centre for Clinical Brain Sciences, University of EdinburghEdinburgh, UK; ^4^Clinical Research Imaging Centre, University of EdinburghEdinburgh, UK

**Keywords:** MRI, neonatal, brain, atlas, parcellation

## Abstract

Neuroimage analysis pipelines rely on parcellated atlases generated from healthy individuals to provide anatomic context to structural and diffusion MRI data. Atlases constructed using adult data introduce bias into studies of early brain development. We aimed to create a neonatal brain atlas of healthy subjects that can be applied to multi-modal MRI data. Structural and diffusion 3T MRI scans were acquired soon after birth from 33 typically developing neonates born at term (mean postmenstrual age at birth 39^+5^ weeks, range 37^+2^–41^+6^). An adult brain atlas (SRI24/TZO) was propagated to the neonatal data using temporal registration via childhood templates with dense temporal samples (NIH Pediatric Database), with the final atlas (Edinburgh Neonatal Atlas, ENA33) constructed using the Symmetric Group Normalization (SyGN) method. After this step, the computed final transformations were applied to T2-weighted data, and fractional anisotropy, mean diffusivity, and tissue segmentations to provide a multi-modal atlas with 107 anatomical regions; a symmetric version was also created to facilitate studies of laterality. Volumes of each region of interest were measured to provide reference data from normal subjects. Because this atlas is generated from step-wise propagation of adult labels through intermediate time points in childhood, it may serve as a useful starting point for modeling brain growth during development.

## Introduction

Labeled atlases provide anatomic information to a range of structural and diffusion MRI (sMRI, dMRI) analysis tasks including structural connectivity mapping and spatio-temporal modeling. In early development such approaches have the potential to provide neuroscientific and clinical advances including: provision of quantitative measures of typical brain growth *in vivo*, so defining “normal” for a newborn population; mapping of atypical trajectories following adverse exposures such as preterm birth; evaluation of tissue effects of neuroprotective treatment strategies that are ready for evaluation in humans; uncovering neural substrates for childhood impairment; and facilitating investigation of the early life origins of adult neurological and psychiatric disease.

The majority of human brain atlases have been developed using adult data (for review see Evans et al., 2012), and their use for studying the brain during early life may not be valid due to differences in adult and newborn anatomy and image properties (Muzik et al., 2000; Wilke et al., 2003; Kazemi et al., 2007; Yoon et al., 2009; Kuklisova-Murgasova et al., 2011). The latter include marked variation in head size and shape, maturational processes leading to changes in signal intensity profiles, relatively lower spatial resolution, and lower contrast between tissue classes (Matsuzawa et al., 2001; Paus et al., 2001; Lenroot and Giedd, 2006; Knickmeyer et al., 2008; Serag et al., 2011; Vardhan et al., 2014). Such differences can lead to misclassification of tissues/structures, so it is essential to match the study group to age-appropriate reference volumes and a number of templates have been developed for this purpose (Sanchez et al., 2012a,b; Fillmore et al., 2015; Richards et al., 2016).

Atlases can be created by manual delineation of a single subject or a small number of subjects. Several investigators have defined protocols to delineate regions of interest (ROIs) in neonatal data. For example, Gilmore and colleagues manually parcellated a single neonatal brain into 16 cortical regions, 20 subcortical regions, brainstem, and cerebellum (Gilmore et al., 2007); Goussias and colleagues manually parcellated 20 neonatal brains (15 preterm and 5 term-born infants) into 50 regions (the ALBERTs atlas; Gousias et al., 2012); and Kabdebon et al. (2014) created a 94 region neonatal single-subject template by adapting an adult brain atlas (Tzourio-Mazoyer et al., 2002), and used it to derive probability maps for the locations of six main sulci in cohort of 16 newborn infants. In recent work, Alexander et al. (2015) manually labeled 33 cortical areas per hemisphere corresponding to those in the Desikan-Killiany adult brain atlas (Desikan et al., 2006) in three term neonates. While such atlases describe anatomical detail well (Gilmore et al., 2007; Kabdebon et al., 2014; Alexander et al., 2015), they may not capture population diversity adequately (Evans et al., 2012), are time-consuming to generate and are susceptible to inter- and intra-rater variability.

Some of these issues can be overcome using computational modeling techniques. For example, the UNC atlas was created using image registration and label fusion to propagate an adult brain atlas to 95 neonates through 2 and 1 year old templates (Tzourio-Mazoyer et al., 2002; Shi et al., 2011). Wu and colleagues used large deformation registration to propagate 62 neuroanatomical labels from adults to 15 neonatal brains and performed multi-atlas labeling based on accurate prior-based tissue segmentation (Wu et al., 2014). Makropoulos and colleagues performed multi-atlas segmentation by label fusion using the ALBERTs atlas (Makropoulos et al., 2014), and subsequently propagated the segmentations (plus labels of cortical ribbon) to the coordinate space of Serag et al. (2012a) and averaged these data with an age kernel at each timepoint to create a 4D atlas with 87 labeled structures (Makropoulos et al., 2016). While these atlases are generally generated from a large cohort and capture population diversity, they are prone to registration error due to shape and tissue contrast differences between adult and neonatal brains.

There are also approaches that combine single subject parcellation with computational methods to create a template. For example, Oishi and colleagues created a template from 20 subjects and propagated a manually labeled single subject (122 regions including white matter parcellations) to the template using image registration (Oishi et al., 2011); and subsequently, Zang and colleagues modified the atlas to represent the average anatomic features of the study group by evolving the initial atlas to the representative “center” of the study population, based on the morphological information (Zhang et al., 2014).

In summary, recent advances in standardized delineation of ROIs and computational modeling have led to the development of templates for studies of childhood brain development. However, most existing neonatal atlases contain less anatomical information compared to adult atlases, often include atypical participants which leaves uncertainty about “normal” representation. They also work mainly with one modality and use labeling protocols that do not map readily to established adult atlases, and none facilitate studies of laterality in early life when it may be desirable to distinguish asymmetries in the study population from those of the atlas. These limitations led us to create a new neonatal atlas (ENA33), which has the following features:

1. ENA33 is generated exclusively from healthy control subjects, so represents “normal.”
2. The atlas has 107 anatomical regions transformed from an adult atlas, so it is consistent with adult label protocols.
3. ENA33 is operable across different modalities including sMRI and dMRI.
4. Symmetric templates are provided to facilitate studies of cerebral laterality.

## Materials and methods

### Overview

The atlas construction framework consists of two main steps. First, each subject is parcellated into anatomical ROIs using temporal registration (Serag et al., 2012b) of an adult atlas (Rohlfing et al., 2010) via intermediate spatio-temporal templates of the National Institutes of Health Pediatric Database (NIHPD; Fonov et al., 2009, 2011). Second, a groupwise atlas is constructed from the parcellated cohort of healthy neonates using Symmetric Group Normalization (SyGN; Avants et al., 2010).

### Participants

Thirty-three healthy infants born at term (>37 weeks' postmenstrual age, PMA) with mean PMA at birth 39^+5^ weeks (range 37^+2^–41^+6^) and with mean birthweight of 3.42 kg (2.35–4.67) were recruited from the Royal Infirmary of Edinburgh, UK, between July 2012 and September 2015. Exclusion criteria were congenital infection, intrauterine growth restriction, major chromosomal abnormalities, evidence of central nervous system malformation or injury on MRI and contraindications to MRI scanning. Underwent MRI at mean 42^+2^ weeks (range 39–47^+1^). Results from a subset of the group have been reported previously (Anblagan et al., 2015). Ethical approval for the study was obtained from the National Research Ethics Service (South East Scotland Research Ethics Committee), and informed written parental consent was obtained for each subject in accordance of the Declaration of Helsinki.

### Image acquisition

A Siemens MAGNETOM Verio 3T MRI clinical (Siemens, Healthcare Gmbh, Erlangen, Germany) and 12-channel Siemens phased-array head matrix coil were used to acquire the following scans: 3D T1-weighted (T1w) MPRAGE (TR = 1650 ms, TE = 2.43 ms, inversion time = 160 ms, flip angle = 9°, acquisition plane = sagittal, voxel size = 1 × 1 × 1 mm, FOV = 256 mm, acquired matrix = 256 × 256, acquisition time = 7 min 49 s and acceleration factor = 2); T2-weighted (T2w) SPACE (TR = 3800 ms, TE = 194 ms, flip angle = 120°, acquisition plane = sagittal, voxel size = 0.9 × 0.9 × 0.9 mm, FOV = 220 mm, acquired matrix = 256 × 218, acquisition time = 4 min 32 s); dMRI using a protocol consisting of 11 T2- and 64 diffusion-weighted (*b* = 750 s/mm^2^) single-shot, spin-echo, echo planar imaging volumes acquired with 2 mm isotropic voxels (TR = 7300 ms, TE = 106 ms, FOV = 256, acquired matrix = 128 × 128, 50 contiguous interleaved slices with 2 mm thickness, acquisition time = 9 min 29 s). To reduce eddy current induced artifacts and shimming errors to a minimum in the dMRI protocol, an optimized bipolar gradient pulse scheme was employed with a manually selected shim box covering a region extending from the top of the head to several centimeters below the chin.

Infants were examined in natural sleep with pulse oximetry and electrocardiography data monitoring. Ear protection was used for each infant comprising earplugs placed in the external ear and neonatal earmuffs (MiniMuffs, Natus Medical Inc., CA).

### Image registration

For each registration between two different images, a linear transformation was first computed and used as an initialisation to compute a non-linear transformation. In other words, a transformation *T*(**x**) for a point **x** in 3D space with coordinates x, y, and z is computed as follows:

(1) $$T\left(x\right)=T_{global}\left(x\right)+T_{local}(x)$$

where *T*_*global*_ represents the linear transformation and *T*_*local*_ represents the non-linear transformation. The computed transformation maps all the points of a “Target” volume to a “Source” volume (*T*_*Target, Source*_).

The interpolation used for all intensity images was B-spline because of its efficacy (Meijering, 2000); and Nearest Neighbor interpolation was used for label maps so as not to introduce new classes.

### Pre-processing

For dMRI, Fractional Anisotropy (FA) and Mean Diffusivity (MD) were calculated using the Camino Diffusion MRI Toolkit (http://cmic.cs.ucl.ac.uk/camino; Cook et al., 2006). For each subject, the T1w volume was selected as the reference anatomy to which the T2w scan was linearly registered (6 degrees of freedom) using NyftiReg (http://cmictig.cs.ucl.ac.uk/research/software/niftyreg; Ourselin et al., 2001; Modat et al., 2010). Then FA and MD were mapped to the same T1w space using the transformation of the first T2w volume of the dMRI dataset (B0) to T1w, using Advanced Normalisation Tools (ANTs, http://stnava.github.io/ANTs; Avants et al., 2008) with mutual information as the similarity metric (Studholme et al., 1999). Intra-subject registration of diffusion maps involved linear plus a non-linear registration, with the aim of minimizing distortions associated with the single-shot spin-echo echo planar imaging acquisition sequence. We used affine and SyN (Avants et al., 2008) with a four-level multi-resolution scheme which ran until convergence or a fixed (maximum) number of iterations was reached. We allowed up to 100 iterations at the first level, 100 iterations at the second level, 100 iterations at the third level and 20 iterations at the full resolution. The rest of the parameters were set to default settings.

A brain mask was computed from the T1w volumes by removing non-brain tissues and skull using the ALFA method (Serag et al., 2016). The resulting mask was applied to all co-registered modalities, and all volumes were corrected for intensity inhomogeneity using the N4 method (Tustison et al., 2010). After the process of label propagation and template creation, all the subjects were affine registered to the 42 weeks template of Serag et al. (2012a).

All the results were checked after this preprocessing to ensure that the N4 method and the skull stripping performed correctly.

### Tissue segmentation

To create tissue segmentations, T1w images were first registered non-linearly to the closest age-matched T1w template from the 4D atlas (Serag et al., 2012c) using Free-Form Deformation (Rueckert et al., 1999) implemented in NiftyReg (Ourselin et al., 2001; Modat et al., 2010) with default parameters. Then, the expectation-maximization (EM) algorithm (Van Leemput et al., 1999; Kuklisova-Murgasova et al., 2011) was used to classify each voxel into a tissue class based on voxel intensity information and spatial-based probabilities (Serag et al., 2012c); after this, segmentations were mapped back to the subject's native space. The tissue probability maps were constructed by averaging the tissue segmentations to produce maps of gray matter (GM), white matter (WM), and cerebrospinal fluid (CSF).

### Temporal registration via spatio-temporal atlases

To parcellate the neonatal brain, the SRI24/TZO adult brain atlas (Rohlfing et al., 2010) with 90 ROIs (cortical and sub-cortical structures only) was propagated to the neonatal template using a spatio-temporal atlas from the online database of the NIHPD (Fonov et al., 2009, 2011) containing age-dependent templates between birth and 4.5 years old (4.5, 3.5, 2.5, 2, 1.5, 1.25, 1, 0.75, 0.5, 0.25, and 0 year [neonate]; McConnell Brain Imaging Centre; http://www.bic.mni.mcgill.ca/ServicesAtlases/NIHPD-obj2). The SRI24/TZO atlas is based on the transformed version of the single-subject Automated Anatomical Labeling (AAL) atlas (Tzourio-Mazoyer et al., 2002) to 24 healthy subjects.

To model the very wide anatomical differences between adult and neonatal brain, we used the LISA method (Serag et al., 2012b) where spatio-temporal atlases are used to aid the registration process between two images taken over large time-interval, as it provides prior information about the missing anatomical evolution between the two images to be registered. Given a pair of structural images from the adult atlas (*I*_*SRI*24_) and our neonatal cohort (*I*_*NEO*_), we aim to find a transformation *T*_*NEO, SRI*24_ that maps every location in *I*_*NEO*_ to *I*_*SRI*24_ by estimating a deformation field to register *I*_*SRI*24_ to *I*_*NEO*_. To do this, we first mapped each template of the NIHPD to the preceding one: *T*_3.5 → 4.5_ = *NIHPD*_3.5_ → *NIHPD*_4.5_, *T*_2.5 → 3.5_ = *NIHPD*_2.5_ → *NIHPD*_3.5_,…,_*T*_0 → 0.25_ = *NIHPD*0_ → *NIHPD*_0.25_. After this step, the adult atlas was mapped to the 4.5 year atlas (*T*_4.5 → *SRI*24_ = *NIHPD*_4.5_ → *SRI*24) and the neonatal NIHPD template to NEO (*T*_0 → *NEO*_ = *NEO* → *NIHPD*_0_). All the transformations were then concatenated together:

(2) $$T_{NEO\to SRI\text{24}}=T_{NEO\to 0}{}^{\circ}T_{0\to \text{0}.\text{25}}{}^{\circ}\ldots \;{}^{\circ}T_{\text{4}.\text{5}\to \text{3}.\text{5}}{}^{\circ}T_{\text{4}.\text{5}\to SRI\text{24}}$$

**Figure 1:**
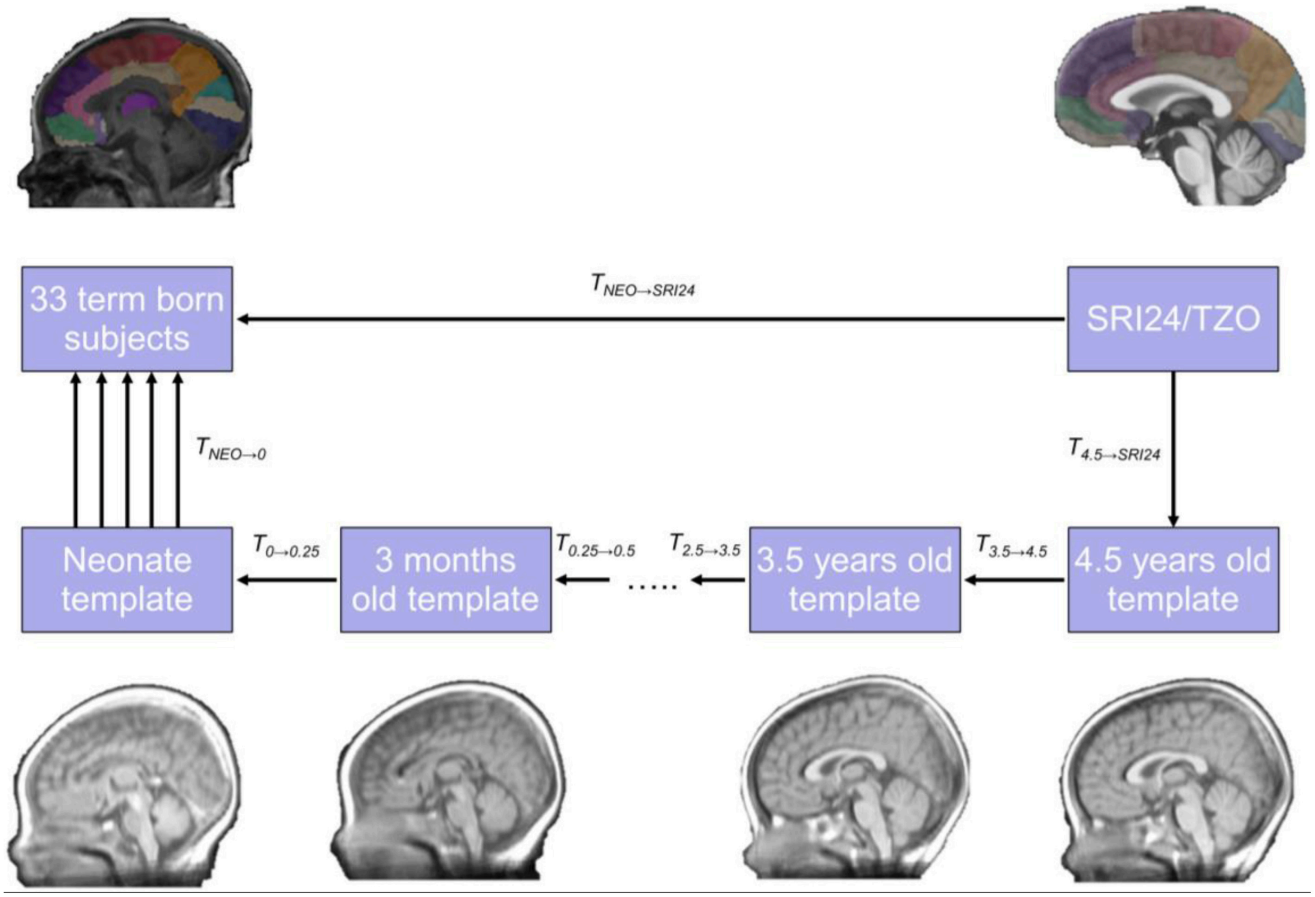
**The framework used for temporal atlas propagation**. The SRI24/TZO adult atlas is propagated to the neonatal template from the NIHPD atlas through intermediate time points, and finally to the cohort under study.

Finally, the combined transformation from the previous step (*T*_*NEO* → *SRI*24_) was used to derive the registration between the *I*_*SRI*24_ and *I*_*NEO*_. The temporal registration process used is summarized in Figure 1.

After registration, the transformation allowed locations in the target image to be mapped to locations in the source image. All the temporal registrations were performed using affine plus SyN (Avants et al., 2008) with mutual information as the similarity metric (Studholme et al., 1999), since this is suitable for contrast changes associated with myelination of the brain during development. The last step, *T*_0 → *NEO*_ = *NEO* → *NIHPD*_0_, was performed using cross correlation (Yoo and Han, 2009), because in this case the neonatal T1w template of the NIHPD was registered to subjects where there was no change in the contrast, so registration is intra-modality.

### Template and atlas construction

For template creation, we used the SyGN. This method works by coupling the intrinsic symmetry of each pairwise registration and optimizing the shape-based sharpening/averaging of the template appearance. The method has been used in previous studies with successful results (Avants et al., 2010, 2015; Zhan et al., 2013).

The SyGN method robustly maps populations to a common space by finding the template and set of transformations that gives the “smallest” parameterization of the dataset (Avants et al., 2010). The metric distance between the average affine transformation and the identity affine transformation as well as the diffeomorphism lengths gives the size of the parametrization. The method may be initialised using an external template or an inital template (Ī) that can be derived from the database of *n* images (*I*_*i*_). In this work, a 42 weeks template (the closest age-matched template to the mean of the cohort under study) from the 4D atlas (Serag et al., 2012a) was used as an initial template.

SyGN optimizes the shape of Ī via a diffeomorphism, ψ (which contains an affine transformation with 12 degrees of freedom), such that the size and shape of the brain converges to the group mean. This is achieved by optimizing the following energy iteratively,

(3) $$E_{\bar{I}}=\underset{i}{\sum }E_{SyN,\prod }(\bar{I},\;I^{i},\phi^{i})\;where\;\forall i,\phi^{i}(x,0)=\;\psi (x)$$

here ψ is a diffeomorphism representing the initial conditions of each optimal transformation (ϕ^*i*^) that maps every point **x** in a 3D space of a image *I*^*i*^) to a reference image (Ī). The solution for each pairwise problem is obtained using SyN (Avants et al., 2008). The algorithm iteratively minimizes the energy $E_{\bar{I}}$ with respect to the set of ϕ^*i*^ through distributed computing (Avants et al., 2011). In this study, all images were previously affine registered to the initial template, so ψ did not contain an affine transformation. The procedure first optimizes the mappings with a fixed template, then, optimizes the template appearance with fixed shape and mappings, and, finally, optimizes the template shape. The process then repeats. The final template is obtained after four iterations.

The final transformations were applied to map the corresponding label maps, tissue segmentation, T2w, FA and MD data to the final template space. To create the final label map majority-voting (Heckemann et al., 2006) of all the propagated labels to the template space was used, because it is known to perform well in studies of neonates (Shi et al., 2011).

Studies of brain laterality benefit from a symmetric atlas because of the challenge of distinguishing asymmetries in the study group from those in atlas space, so we created a symmetric version of the atlas. This was created by flipping each subject's T1w volume left to right, and using each volume as an independent subject in the template creation. The final transformations were then applied to the other modalities which were also flipped including the label maps, using methods described by Fonov et al. (2011, 2009). To create the final symmetric label map majority-voting (Heckemann et al., 2006) was also used.

An additional color map to the standard coding scheme of the SRI24/TZO was created using brainCOLOR (Klein et al., 2010) to aid visualization of lobes. This computes the optimal color assignments for regions in a 2D or 3D brain image using a brute force strategy to maximize the distinguishability of adjacent regions while simultaneously choosing perceptually similar colors for groups of regions.

### Validation

Cross-correlation (CC) between registration of consecutive time points of the spatio-temporal atlas was used to evaluate accuracy of the final registration using methods described for temporal modeling of perinatal MRI data (Serag et al. (2012c).

After temporal propagation, labels were inspected and edited where necessary by a radiologist experienced in neonatal brain MRI (A.G.W.) according to the protocols defined in the *The Human Brain During the Third Trimester* (Bayer and Altman, 2003) using ITK-SNAP (http://www.itksnap.org; Yushkevich et al., 2006). After the template was created, all labels were re-checked according to the same protocol.

The accuracy of registration used for label propagation between the subjects and the registered atlas was tested. To do this, five landmarks were placed in ten randomly selected subjects and the atlas; the atlas was then registered to the subjects using affine and SyN (Avants et al., 2008) using cross-correlation as the similarity metric. The Euclidean distance between the landmarks of the subjects and those of the registered atlas were measured (Black et al., 2001; McLaren et al., 2009; Ella and Keller, 2015; Love et al., 2016). The landmarks were placed at: the most rostral point of right and left superior temporal gyrus viewed in the coronal plane at the level of the third ventricle (referred to as cortical left and right in **Table 2**); the wall of the right and left bodies of the lateral ventricles at the level of the third ventricle in coronal plane (referred to as ventricles left and right in **Table 2**); and the floor of the fourth ventricle in the sagittal plane (referred as cerebellum in **Table 2**. To investigate potential bias due to intra- and inter-rater variability in landmark placement, landmarks were placed by the same rater twice and by another rater. Raw measurements and intraclass correlation coefficient (ICC) using a two way mixed effects model are reported.

To evaluate agreement of volumetric measurements obtained from ENA33 with those of a comparable atlas,(the UNC atlas, which is derived from the same adult atlas), we compared lobar volumes using the protocol described by Tzourio-Mazoyer et al. (2002): *Central Region, Frontal Lobe, Temporal Lobe, Parietal Lobe, Occipital Lobe, Limbic Lobe, Insula and Sub Cortical Gray Nuclei* plus the *Corpus Callosum, Lateral Ventricles*, the *Brainsteam* and the *Cerebellum*. The proportion of intra-cranial volume of each region was calculated. Both label maps were multiplied by the respective mask, then the lobular volume was divided by the brain volume (mask volume).

To investigate differences between the asymmetric and symmetric versions an Asymmetry coefficient (*S*) was calculated. The coefficinet (*S*) is defined as:

(4) $$S=\frac{2*\left|V_{L}-V_{R}\right|}{V_{L}+V_{R}}$$

where *V*_*L*_ is the volume of the left ROI and *V*_R_ is the volume of right ROI. The main difference with the index defined in previous studies (Luders et al., 2004; Dubois et al., 2010) is that, originally, the index is defined to perform voxel-wise studies, and here it is adapted to a volumetric analysis. If template construction and label fusion were completely error free across the volume then *S* would have a value of 0 for all regions in the symmetric version.

### Volumetric analysis

We non-linearly registered the final atlas to all subjects using ANTs (Avants et al., 2008) with the same parameters as above, with the aim of calculating the volume and different dMRI metrics of all ROIs for both hemispheres of the brain. The volumes and dMRI metrics for each region were calculated using FSL (http://fsl.fmrib.ox.ac.uk; Jenkinson et al., 2012).

## Results

### Neonatal brain parcellation

**Figure 2:**
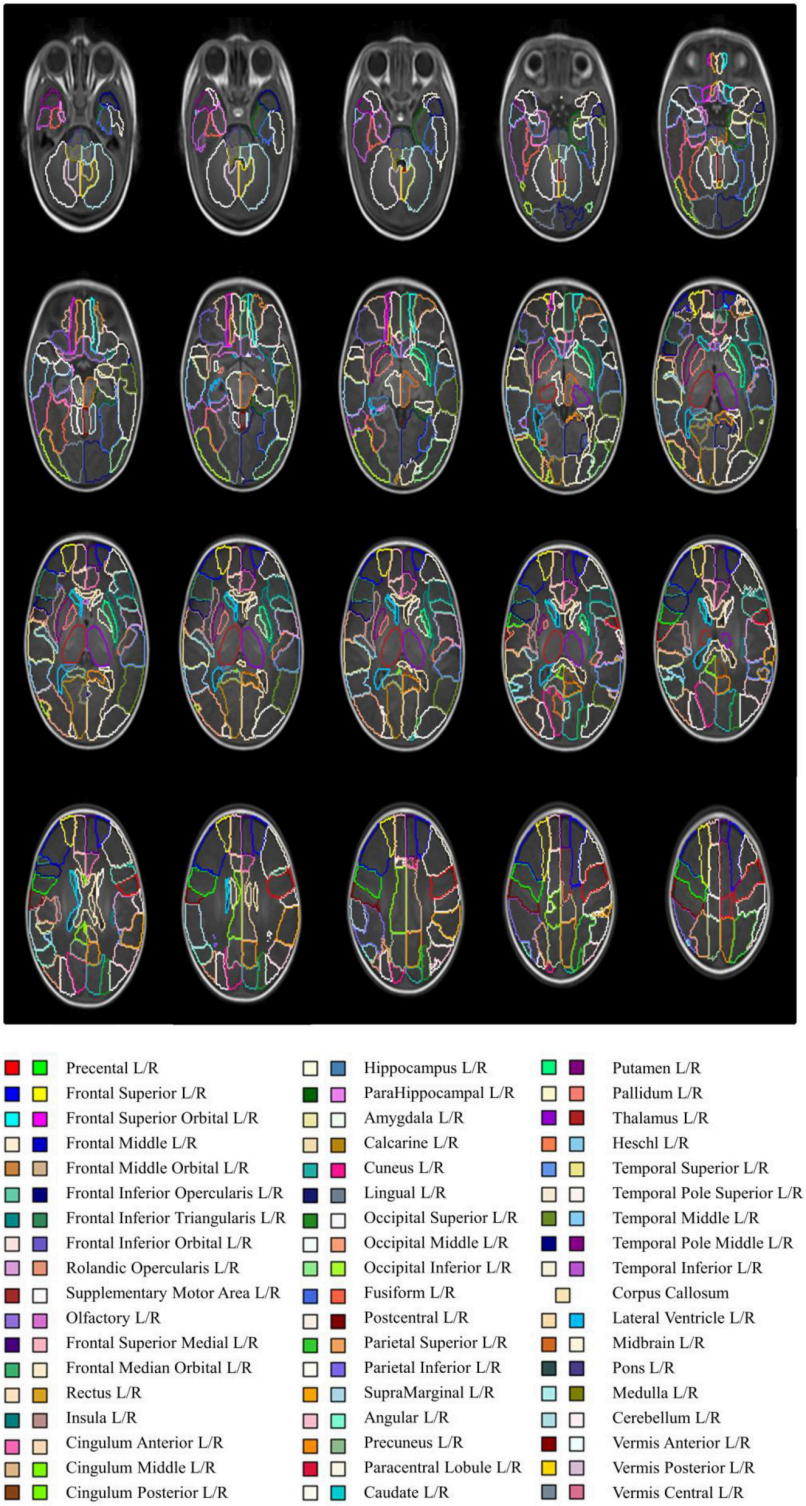
**Anatomical parcellation of the neonatal brain (axial view)**. The slices have 3 mm distance.

**Figure 3:**
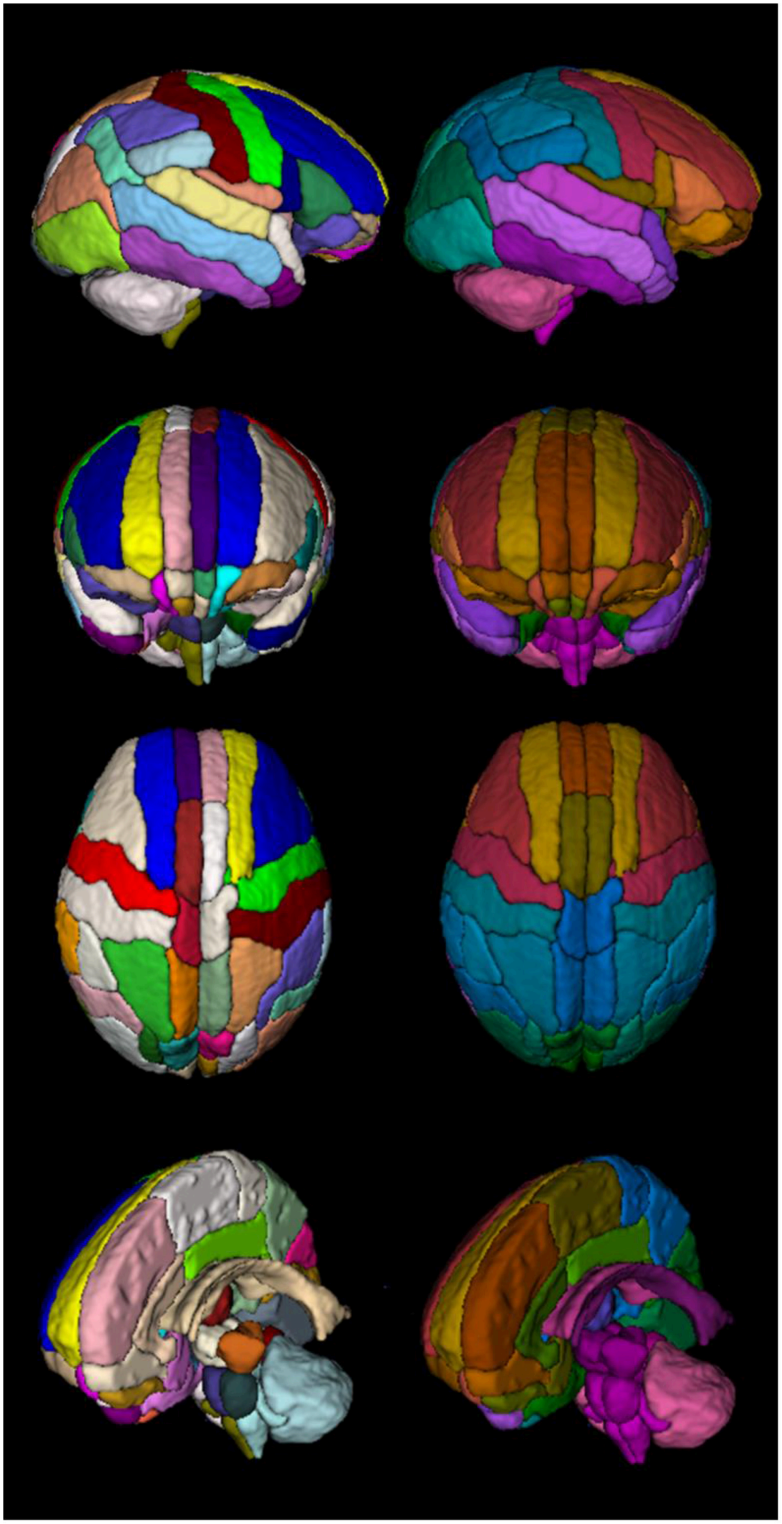
**3D rendered of the atlas comparing both color codes: standard color code (left column) vs. created color code (right column)**.

ENA33 is shown in transverse sections using default color scheme (generated by ITK-SNAP) in Figure 2, and Table 1 lists the labels. The 3D volume rendered atlas is shown in Figure 3., using the default and brainCOLOR color coding generated schemes.

**Table 1:** **Anatomical definition of all the ROIs and the correspondent Labe ID**.

**Anatomical definition**	**Label ID**	**Anatomical definition**	**Label ID**
Precentral left	1	Fusiform left	55
Precentral right	2	Fusiform right	56
Frontal superior left	3	Postcentral left	57
Frontal superior right	4	Postcentral right	58
Frontal superior orbital left	5	Parietal superior left	59
Frontal superior orbital right	6	Parietal superior right	60
Frontal middle left	7	Parietal inferior left	61
Frontal middle right	8	Parietal inferior right	62
Frontal middle orbital left	9	Supramarginal left	63
Frontal middle orbital right	10	Supramarginal right	64
Frontal inferior opercularis left	11	Angular left	65
Frontal inferior opercularis right	12	Angular right	66
Frontal inferior triangularis left	13	Precuneus left	67
Frontal inferior triangularis right	14	Precuneus right	68
Frontal inferior orbital left	15	Paracentral lobule left	69
Frontal inferior orbital right	16	Paracentral lobule right	70
Rolandic opercularis left	17	Caudate left	71
Rolandic opercularis right	18	Caudate right	72
Supplementary motor area left	19	Putamen left	73
Supplementary motor area right	20	Putamen right	74
Olfactory left	21	Pallidum left	75
Olfactory right	22	Pallidum right	76
Frontal superior medial left	23	Thalamus left	77
Frontal superior medial right	24	Thalamus right	78
Frontal median orbital left	25	Heschl left	79
Frontal median orbital right	26	Heschl right	80
Rectus left	27	Temporal superior left	81
Rectus right	28	Temporal superior right	82
Insula left	29	Temporal pole superior left	83
Insula right	30	Temporal pole superior right	84
Cingulum anterior left	31	Temporal middle left	85
Cingulum anterior right	32	Temporal middle right	86
Cingulum middle left	33	Temporal pole middle left	87
Cingulum middle right	34	Temporal pole middle right	88
Cingulum posterior left	35	Temporal inferior left	89
Cingulum posterior right	36	Temporal inferior right	90
Hippocampus left	37	Corpus callosum	91
Hippocampus right	38	Lateral ventricle left	92
Parahippocampal left	39	Lateral ventricle right	93
Parahippocampal right	40	Midbrain left	94
Amygdala left	41	Midbrain right	95
Amygdala right	42	Pons left	96
Calcarine left	43	Pons right	97
Calcarine right	44	Medulla left	98
Cuneus left	45	Medulla right	99
Cuneus right	46	Cerebellum left	100
Lingual left	47	Cerebellum right	101
Lingual right	48	Vermis anterior left	102
Occipital superior left	49	Vermis anterior right	103
Occipital superior right	50	Vermis posterior left	104
Occipital middle left	51	Vermis posterior right	105
Occipital middle right	52	Vermis central left	106
Occipital inferior left	53	Vermis central right	107
Occipital inferior right	54		

An intial step from adult to 4.5 years was used because we did not find that any additional benefit was conferred by the inclusion of three time points at 15.5, 10.5, and 6.5 years: the normalized cross correlation between registered images generated using both approaches was ≈0.98.

### Application of the atlas to multi-modal data

**Figure 4:**
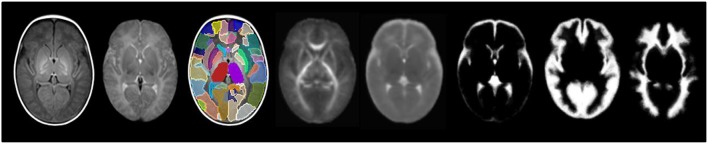
**From left to right: T1w template, T2w template, label parcellation map overlaid on T1w template, FA template, MD template and tissue probability maps for CSF, GM, and WM**.

Figure 4 shows the templates for available modalities. Nine participants had T2w volumes that were free of motion artifact and suitable for registration, so the T2w template shown is constructed from a subset.

### Validation

Cross correlation between the intermediate time points was high (0.93 ± 0.05). The smallest values were 0.81 and 0.87 for the last two steps (3 month to 1 months and 6 months to 3 months), which is a period of dynamic change in signal intensity associated with myelination. The rest of the values were above 0.94.

Assessment of parcellations by an expert (A.G.W.) according to a reference atlas (*The Human Brain During the Third Trimester;* Bayer and Altman, 2003) led to minor edits in thalamus, pallidum, and putamen bilaterally. The following labels were added manually: *posterior fossa* (with its corresponding sub-areas), *lateral ventricles* and *corpus callosum*. After the template was created, the labels were checked again, and minor corrections were made at the brain–CSF boundary only.

The Euclidean distance between landmarks in native space and those of the registered atlas were in an acceptable range for both raters (Table 2), and the ICC was >0.95 for intra- and inter-rater variation.

**Table 2:** **Landmark registration accuracy (Euclidean distance between ENA33 and individuals)**.

	**Distance (mm)**		
	**Rater 1**	**Rater 1 second time**	**Rater 2**
**Landmark**	**Mean (SD)**	**Mean (SD)**	**Mean (SD)**
Cortical left	1.29 (0.82)	1.17 (0.79)	1.66 (0.81)
Cortical right	1.58 (0.91)	1.58 (0.86)	1.89 (1.21)
Cerebellum	1.15 (0.59)	1.02 (0.59)	1.16 (0.61)
Lateral ventricle left	1.89 (0.49)	1.86 (0.59)	2.33 (0.60)
Lateral ventricle right	1.77 (0.63)	1.85 (0.92)	2.08 (0.85)

There was broad agreement between the lobar volumes calculated as a proportion of intracranial volume from ENA 33 and the UNC atlas, shown in Table 3.

**Table 3:** **Volumes of interest calculated from ENA33 and UNC atlases**.

	**ENA 33 Atlas**	**UNC Atlas**
**Region**	**Proportion of intracranial volume (%)**	**Proportion of intracranial volume (%)**
Central region	7.57	7.46
Frontal lobe	34.41	36.3
Temporal lobe	8.80	11.19
Parietal lobe	8.53	11.6
Occipital lobe	13.23	14.55
Limbic lobe	5.9	8.57
Insula	1.46	1.74
Sub cortical gray nuclei	3.53	3.75
Corpus callosum	1.06	–
Lateral ventricles	0.55	–
Brainsteam	1.98	–
Cerebellum	5.58	–

### Analysis of normative data from 33 healthy newborns

Labels were propagated to the images of the 33 healthy infants to provide reference sMRI data for each ROI. Table 4 shows the mean volumes for all ROIs.

**Table 4:** **Volumes for all brain regions**.

**Region**	**Right hemisphere Mean (SD)/cm^3^**	**Left hemisphere Mean (SD)/cm^3^**
Precental	8.87 (1.19)	9 (1.17)
Frontal superior	9.96 (1.54)	8.59 (1.26)
Frontal superior orbital	1.51 (0.22)	1.4 (0.22)
Frontal middle	11.6 (1.74)	13.27 (1.61)
Frontal middle orbital	2.36 (0.37)	2.56 (0.41)
Frontal inferior opercularis	2.77 (0.36)	2.71 (0.44)
Frontal inferior triangularis	2.89 (0.56)	3.03 (0.57)
Frontal inferior orbital	5.32 (0.78)	4.23 (0.66)
Rolandic opercularis	5.37 (0.74)	3.67 (0.54)
Supplementary motor area	5.81 (0.74)	5.33 (0.77)
Olfactory	1.25 (0.16)	1.31 (0.2)
Frontal superior medial	5.96 (1.04)	6.84 (1.12)
Frontal median orbital	1.47 (0.28)	1.47 (0.3)
Rectus	1.17 (0.2)	1.13 (0.17)
Insula	3.67 (0.28)	3.46 (0.28)
Cingulum anterior	2.47 (0.27)	2.38 (0.32)
Cingulum middle	3.53 (0.4)	3.85 (0.42)
Cingulum posterior	0.35 (0.07)	0.44 (0.08)
Hippocampus	2.08 (0.18)	2.2 (0.18)
Parahippocampal	2.58 (0.22)	2.41 (0.23)
Amygdala	0.65 (0.06)	0.76 (0.06)
Calcarine	4.26 (0.55)	3.8 (0.57)
Cuneus	3.72 (0.51)	3.55 (0.47)
Lingual	7.35 (0.69)	7.83 (0.89)
Occipital superior	2.24 (0.34)	3.45 (0.38)
Occipital middle	5.7 (0.74)	6.43 (0.71)
Occipital inferior	3.34 (0.43)	5.52 (0.53)
Fusiform	5.73 (0.74)	5.06 (0.54)
Postcentral	6.82 (0.98)	7.08 (0.91)
Parietal superior	5.72 (0.73)	4.65 (0.57)
Parietal inferior	2.42 (0.33)	5.75 (0.8)
Supramarginal	3.81 (0.54)	2.98 (0.51)
Angular	3.47 (0.55)	2.25 (0.42)
Precuneus	7.17 (0.69)	7.14 (1.03)
Paracentral lobule	2.17 (0.29)	3.2 (0.45)
Caudate	1.03 (0.12)	1.1 (0.14)
Putamen	1.29 (0.13)	1.57 (0.17)
Pallidum	1.56 (0.24)	1.7 (0.24)
Thalamus	3.81 (0.24)	3.89 (0.28)
Heschl	1.04 (0.24)	0.94 (0.17)
Temporal superior	6.47 (0.69)	7.24 (0.69)
Temporal pole superior	2.65 (0.34)	2.91 (0.35)
Temporal middle	7.46 (0.82)	8.61 (1.02)
Temporal pole middle	1.53 (0.29)	1.35 (0.23)
Temporal inferior	8.24 (0.86)	6.38 (0.78)
Lateral ventricle	2.58 (0.6)	2.75 (0.77)
Midbrain	1.88 (0.11)	1.86 (0.1)
Pons	0.83 (0.1)	1.03 (0.12)
Medulla	2.24 (0.2)	2.57 (0.2)
Cerebellum	11.24 (1.28)	11.17 (1.33)
Vermis anterior	0.8 (0.16)	0.82 (0.18)
Vermis posterior	2.03 (0.4)	1.97 (0.36)
Vermis central	0.69 (0.09)	0.85 (0.13)
Corpus callosum	2.63 (0.36)	

**Figure 5:**
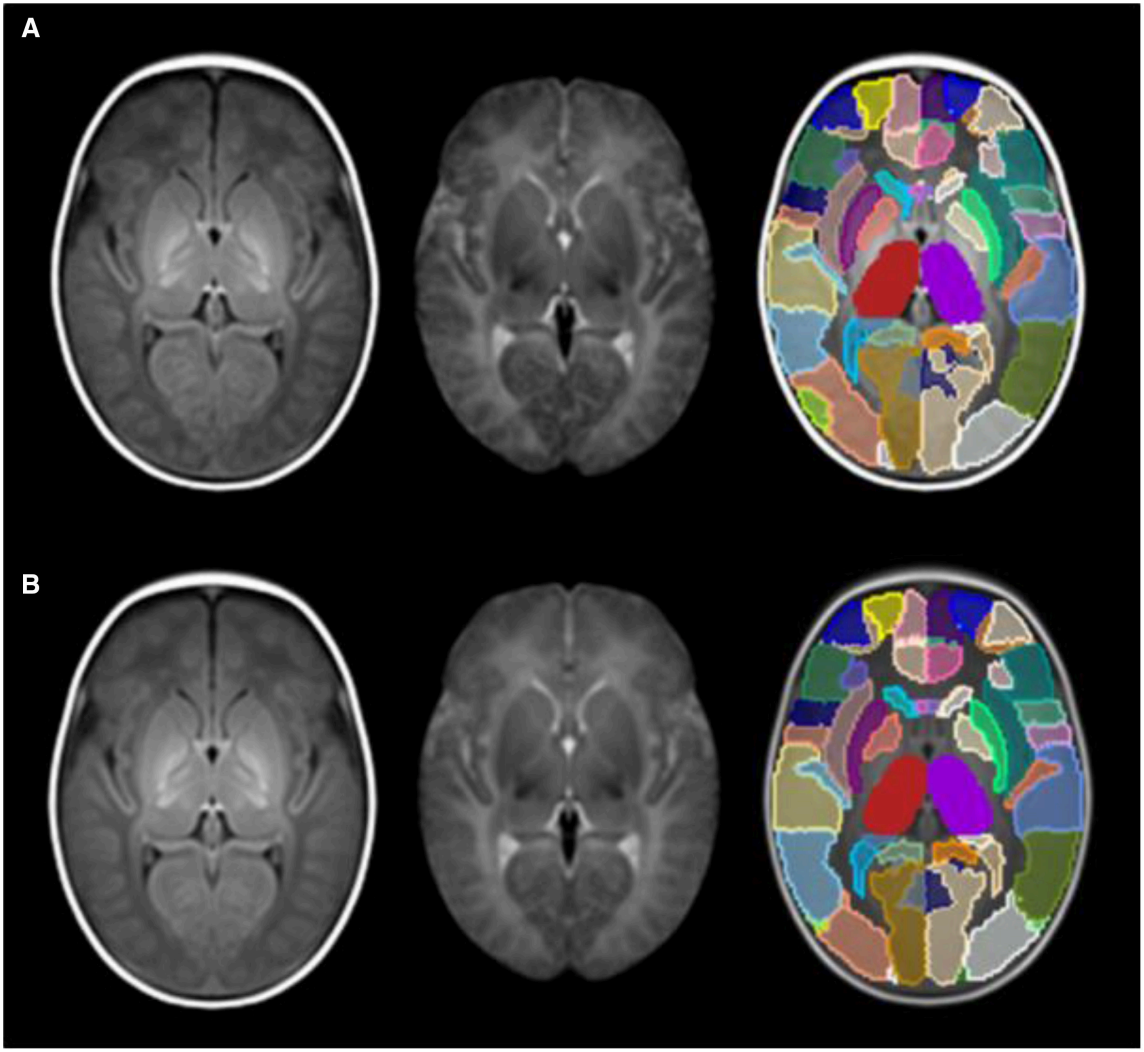
**(A)** asymmetric version of the atlas; **(B)** symmetric version of the atlas. From left to right: T1w template, T2w template and label parcellation map overlaid on T1w template.

**Figure 6:**
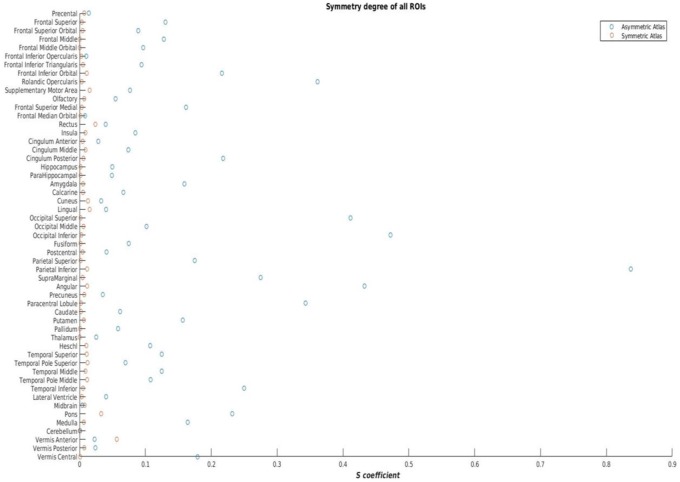
**Asymmetry coefficient in the asymmetric and the symmetric versions of ENA33**.

Figure 5 shows the symmetric version of the atlas compared with the asymmetric version, and Figure 6 shows the differences in S for each ROI. S was < 0.05 for all regions in the symmetric version, and values ranged from 0.01 to 0.9 in the asymmetric version.

## Discussion

Using MRI data from 33 healthy newborn infants, we created a neonatal brain atlas that parcellates the brain into 107 anatomical regions that can be applied to T1w, T2w, dMRI (FA and MD), and tissue probability maps; it also contains a symmetric version of all templates. The framework for atlas creation was based on temporal propagation of a labeled adult brain atlas (SRI24/TZO) via a sequence of MRI templates from childhood to early infancy, which may make it suitable for modeling human brain growth using a consistent set of labels over time. The basis for considering that one-to-one mapping of adult to neonatal structures would be feasible stems from the consistent observation that human cortical gyrification is established during the third trimester of pregnancy, so such that the “adult” configuration is present in the healthy infant born at full term, and this can be discerned using MRI (Armstrong et al., 1995; van der Knaap et al., 1996; Pienaar et al., 2008; Shi et al., 2010).

The SRI24/TZO atlas was used because it represents brain anatomy in an unbiased population-averaged coordinate system, and at the same time, provides a large number of structures in crisp definition so is suitable for label propagation (Rohlfing et al., 2010). An intitial temporal registration step from adult to 4.5 years was used because we did not find additional benefit conferred by the inclusion of three time points at 15.5, 10.5, and 6.5 years.

There is inverted contrast of WM and GM signal between neonatal and adult brain images, which might suggest that the ideal registration between adult and neonatal templates should be performed between neonatal T1w and adult T2w images. However, the use of intermediate templates avoids marked step-wise changes in contrast and it was possible to achieve accurate temporal registration using T1w images with mutual information as the similarity metric (Serag et al., 2012b). The diffeomorphic registration algorithm described by Avants et al. (2008) was used because of its accuracy as demonstrated in a recent comparison of non-rigid registration techniques (Klein et al., 2009), but other algorithms may also be suitable for this framework, including Free-Form Deformation (Rueckert et al., 1999), Large Deformation Diffeomorphic Metric Mapping (LDDMM; Beg et al., 2005), or FNIRT (Jenkinson et al., 2012) among others. For multi-modality template construction, the SyGN framework was used, because of its ability to produce population-specific templates. The main advantage of the method is that it iteratively optimizes the template appearance and template shape (Avants et al., 2015).

The validation strategy we used was both qualitative and quantitative. The requirement for manual editing by an expert according to protocols defined in the *The Human Brain During the Third Trimester* (Bayer and Altman, 2003) was limited to a small number of ROIs. We tested accuracy of temporal registration using cross-correlation and results demonstrated high accuracy of the registration approach (mean CC of 0.93). To confirm accuracy of label propagation we used a landmark approach, and found Euclidean distances in an acceptable range for landmarks selected to represent the cortex, ventricular system and cerebellum. Both intra- and inter-rater variability were low, and the magnitude of difference is likely to be acceptable for most applications.

The volumes reported in this cohort of normal infants are of similar magnitude and variance to those reported in other smaller studies of healthy newborns, albeit at the level of tissue class or larger regions interest, rather than corresponding ROIs (Inder et al., 2005; Boardman et al., 2007; Thompson et al., 2011). We found very similar measurements of lobar volumes as a proportion of ICV between ENA33 and the UNC atlas, which uses a similar label protocol to ENA33. The small differences between the two atlases could be due to the propagation approach (in this work more time points were used, and an extra registration step is implemented), to differences in template construction method and/or to the manual corrections, or they could reflect normal population variation. Further studies that include large numbers of participants with sharing of data and protocols from multiple centers will be required to determine the extent to which small differences in measured values represent population diversity vs. methodological variation.

It should be noted that partial volume effects are not significant for measures derived from the structural volumes with voxel size of ~1 mm^3^ because the template is resampled from 0.86 to 1 mm^3^ (Ashburner and Friston, 2000; Antonova et al., 2005; Serag et al., 2012c). For acquisitions with larger voxel sizes (for example dMRI with 2 mm^3^) it is possible that partial volume effects could confound the extracted metrics.

A beneficial feature of ENA33 is provision of a symmetric version with labels, which is novel for a neonatal populations and could have utility for future study designs involving neonatal data that require identification of asymmetry in the study group. The asymmetry coefficient (*S*) was < 0.05 for all structures in the symmetric atlas but ranged from 0.01 to 0.9 in the asymmetric atlas, which reflects the wide regional variation and the magnitude of asymmetry in healthy newborn brain.

The atlas could be used for different voxel-wise studies or multi-modal applications that are substantially improved by the use of a specific neonatal template, including voxel-based techniques such as Tract-based Spatial Statistics (TBSS; Smith et al., 2006; Ball et al., 2010), Statistical Parametric Mapping (SPM; Ashburner and Friston, 2000), structural connectivity and network analyses or volumetric studies. This atlas can be used to perform studies of laterality when it is important to distinguish template asymmetries from those of the study population.

## Conclusion

In this work, we present a new framework for atlasing the brain in early life. The resulting atlas (ENA33) contains 107 regions with high spatial definition which can be applied to give anatomical context to T1w, T2w volumes, and FA and MD data, whilst also providing tissue probability maps. The way of generating the labels of ENA33 using step-wise propagation of adult labels through intermediate time points makes the atlas consistent with adult atlases, which is very useful in future studies from birth to adulthood. A symmetric version of the atlas is also generated for studies of laterality in the developing brain. The atlas is available to the research community from: http://brainsquare.org, and the raw data from the Brain Images of Normal Subjects (BRAINS) repository (http://www.brainsimagebank.ac.uk; Job et al., 2016).

## Author contributions

MB, AS, MB, and JB designed the work; and MB, AS, JB, and GW analyzed the data. Participants were recruited and images were acquired by DA, ET, RP, SS, GM, SS, and JB. MB wrote the first draft and all authors revised the final version critically for important intellectual content. All authors approved the final submitted version, and agree to be accountable for its content.

## Funding

This study was funded by Theirworld, NHS Research Scotland, and NHS Lothian Research and Development.

### Conflict of interest statement

The authors declare that the research was conducted in the absence of any commercial or financial relationships that could be construed as a potential conflict of interest.
